# Winemaking and Bioprocesses Strongly Shaped the Genetic Diversity of the Ubiquitous Yeast *Torulaspora delbrueckii*

**DOI:** 10.1371/journal.pone.0094246

**Published:** 2014-04-09

**Authors:** Warren Albertin, Laura Chasseriaud, Guillaume Comte, Aurélie Panfili, Adline Delcamp, Franck Salin, Philippe Marullo, Marina Bely

**Affiliations:** 1 Univ. de Bordeaux, ISVV, EA 4577, Unité de recherche Œnologie, Villenave d'Ornon, France; 2 Biolaffort, Bordeaux, France; 3 INRA, UMR Biodiversité Gènes et Ecosystèmes, PlateForme Génomique, Cestas, France; University of Strasbourg, France

## Abstract

The yeast *Torulaspora delbrueckii* is associated with several human activities including oenology, bakery, distillery, dairy industry, etc. In addition to its biotechnological applications, *T. delbrueckii* is frequently isolated in natural environments (plant, soil, insect). *T. delbrueckii* is thus a remarkable ubiquitous yeast species with both wild and anthropic habitats, and appears to be a perfect yeast model to search for evidence of human domestication. For that purpose, we developed eight microsatellite markers that were used for the genotyping of 110 strains from various substrates and geographical origins. Microsatellite analysis showed four genetic clusters: two groups contained most nature strains from Old World and Americas respectively, and two clusters were associated with winemaking and other bioprocesses. Analysis of molecular variance (AMOVA) confirmed that human activities significantly shaped the genetic variability of *T. delbrueckii* species. Natural isolates are differentiated on the basis of geographical localisation, as expected for wild population. The domestication of *T. delbrueckii* probably dates back to the Roman Empire for winemaking (∼1900 years ago), and to the Neolithic era for bioprocesses (∼4000 years ago). Microsatellite analysis also provided valuable data regarding the life-cycle of the species, suggesting a mostly diploid homothallic life. In addition to population genetics and ecological studies, the microsatellite tool will be particularly useful for further biotechnological development of *T. delbrueckii* strains for winemaking and other bioprocesses.

## Introduction

The remarkable physiological properties of yeasts have led to their wide use in the field of biotechnology. Their fermentative ability has been exploited by humans for millennia to ferment and preserve beverages and food. The most famous yeast is undoubtedly *Saccharomyces cerevisiae*, which has been making bread, beer [1], wine [2] and spirits [3] -the oldest applications- for ages, and more recently bioethanol [4]. Indeed, population genetics of *S. cerevisiae* reveals a strong connection to human civilization and history, with genetic clustering coinciding with biotechnological applications [5]. This indicates that over hundreds or even thousands of years, human uses promoted the adaptation of *S. cerevisiae* to various food/beverage systems, a process called domestication [6].

Besides *Saccharomyces cerevisiae*, several dozen yeast species are involved in various biotechnological processes, such as *Ogataea angusta* (formerly *Hansenula polymorpha*) for production of recombinant proteins among which pharmaceuticals [7], *Komagataella* (*Pichia*) *pastoris* for production of pharmaceutical/nutrient compounds [8], *Kluyveromyces lactis* var. *lactis* for enzymatic production [9]. However, there are far less data regarding population studies of so-called “non-conventional” yeasts. Indeed, while there are several examples of adaptation of molds and lactic acid bacteria to anthropic food environments [6], it is still unclear to what extent the domestication process shaped yeast evolution.

In this work, we considered a non-conventional yeast species of technological interest, *Torulaspora delbrueckii*. *T. delbrueckii* has been associated with winemaking for decades [10]–[12] and isolated either from grape, must or wine. Although *T. delbrueckii* is generally unable to complete alcoholic fermentation (*i.e.* to consume all sugars), it produces relatively high ethanol concentrations for a non-*Saccharomyces* yeast [11], [13]–[15]. This explains why *T. delbrueckii* was formerly classified within the *Saccharomyces* genus (under *S. rosei* or *S. roseus* name). *T. delbrueckii* also produces low levels of undesirable volatile compounds (hydrogen sulphide, volatile phenols) [16], [17], reduces volatile acidity in high-sugar fermentations when associated with *S. cerevisiae* in mixed cultures [17] and increases sensorial complexity [18]–[20]. Due to its oenological interest, the duo formed by *S. cerevisiae* and *T. delbrueckii* is now becoming a model for studying interaction mechanisms between yeast populations [21]–[23].

Besides its potential for winemaking, *T. delbrueckii* has biotechnological applications in the bread industry [24] due to dough leavening ability associated with high freezing and osmotic tolerance [25]–[27]. *T. delbrueckii* is frequently described as a major component of yeast biota from dough carried over from previous bread making in rural regions [24] or from artisanal bakeries [28]. Commercial exploitation of this species has recently begun, with some *T. delbrueckii* strains that are commercialized in Japan for frozen dough applications [24].

*T. delbrueckii* is also naturally associated with several other human bioprocesses, ranging from food fermentations of silage, cocoa [29], [30], olive [31] or cucumber [32], [33], to distilled and traditional fermented beverage production including mezcal [34], colonche [35], tequila [36], cider [37], strawberry tree fruits juice [38], sugarcane juice [39], [40] and kefir [41]. *T. delbrueckii* is a frequent component of dairy products' microflora, either as desirable ferment for traditional cheeses [42] and fermented milk [43], or as spoilage yeast [44], [45]. Other processed products like soft drinks (fruit juices, etc.) can be spoiled by yeasts including *T. delbrueckii* [46].

In most bioprocesses where *T. delbrueckii* is identified, the species is not added deliberately, unlike *S. cerevisiae* which is usually added as lyophilized inoculum. *T.delbrueckii* naturally colonized a wide range of anthropized habitats. This species is also frequently isolated from natural environments, ranging from soils [47], to plants [48], fruits [49] and insects [50], [51]. Finally, this species, although not considered to be a human pathogen, is occasionally found as a clinical isolate [52] where it is usually referred as *Candida colliculosa*, the anamorphic form of *T. delbrueckii* [53].

Thus, *Torulaspora delbrueckii* is a remarkably ubiquitous yeast species with both natural reservoirs and habitats associated with human activities (winemaking and other bioprocesses), and appears to be a perfect yeast model to search for evidence of domestication besides the baker's yeast *S. cerevisiae*. However, to date, few tools for molecular characterization of *T. delbrueckii* strains were available. Mitochondrial RFLP appears too poorly discriminant at the strain level [24]. Restriction endonuclease analysis associated with pulse-field gel electrophoresis (REA-PFGE), although discriminant [16], is time-consuming and does not allow for accurate population genetics or ecological studies. In this work, we developed eight microsatellite markers for the *T. delbrueckii* species. This new tool was used for the genotyping of 110 strains from various geographical regions and various substrates. We show that the genetic variability of the ubiquitous yeast *T. delbrueckii* is strongly shaped by human activities and in particular by winemaking and other bioprocesses.

## Materials and Methods

### Yeast strains and culture conditions

**Figure 1:**
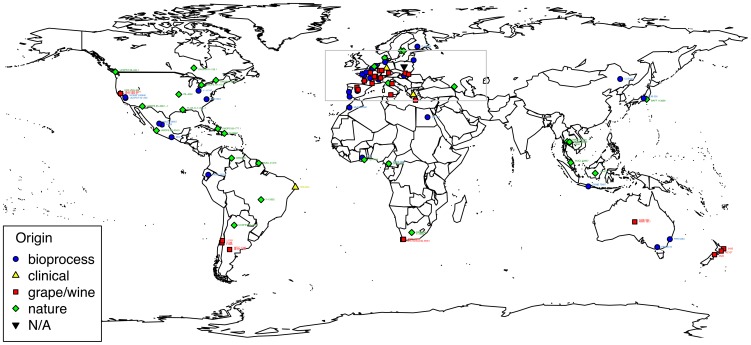
Geographical localisation of the *T. delbrueckii* strains used in this study. 3 strains (CLIB 230, CLIB 503, MUCL 27828) are not represented since their precise isolation location is unknown. More details on European isolates (grey box) can be found on Figure S1.

One hundred and ten strains of *Torulaspora delbrueckii* were sampled from various isolation substrates (grape/wine, nature, clinical, bakery, spoiled food, fermented beverages, dairy products and other bioprocesses) and from worldwide locations (Figure 1, Table S1 and Figure S1). In addition, the type strains of *T. franciscae, T. pretoriensis, T. microellipsoides, T. globosa, T. indica* [54], *T. maleeae* [55], and *T. quercuum* [56] were used to test whether the microsatellites developed for *T. delbrueckii* could be useful for other *Torulaspora* species.

All strains were grown at 24°C in YPD-based medium containing 1% yeast extract (w/v, Difco Laboratories, Detroit, MI), 1% Bacto peptone (w/v, Difco), and 6% glucose (w/v), supplemented or not with 2% agar (w/v). For a quick assessment of respiratory-ability, cells were plated on YPGly medium, containing glycerol as unique source of carbon (1% yeast extract (w/v, Difco), 1% Bacto peptone (w/v, Difco), 2% (v/v) glycerol and 2% (w/v) agar). A minimum medium SD containing 0.67% Yeast Nitrogen Base (w/v, Difco), 2% glucose (w/v) and 2% agar (w/v) was used to test for prototrophy/auxotrophy. The ability to sporulate was checked by microscopy after 3 days at 24°C on acetate medium (1% potassium acetate, 2% agar). *T. delbrueckii* strains usually formed asci with one unique ascospore, while ascii with two to four spores were more rare.

### Genomic DNA extraction

For DNA extraction, cells grown on YPD medium were lysed using a FastPrep-24 instrument (MP Biomedicals, Illkirch, France): 100 μL of glass beads (acid-washed, 425–600 μm, Sigma, Lyon, France) were added to cells pellet as well as 300 μl od Nuclei Lysis solution (Wizard Genomic DNA purification Kit, Promega). Cells were crushed through 2 cycles of 20 s (max. speed). Subsequent DNA extraction was performed with the Wizard Genomic DNA purification Kit (Promega) following the manufacturer's protocol.

### Species assessment

PCR-RFLP of the ITS region (with *Eco*RI digestion) was performed as described by Granchi et al. [57] to confirm the *Torulaspora* genus. In addition, we developed two additional PCR-RFLP markers to discriminate *T. delbrueckii* strains from the other *Torulaspora* species: briefly, we amplified D1/D2 domain by means of universal primers NL1 and NL4 [58].

**Figure 2:**
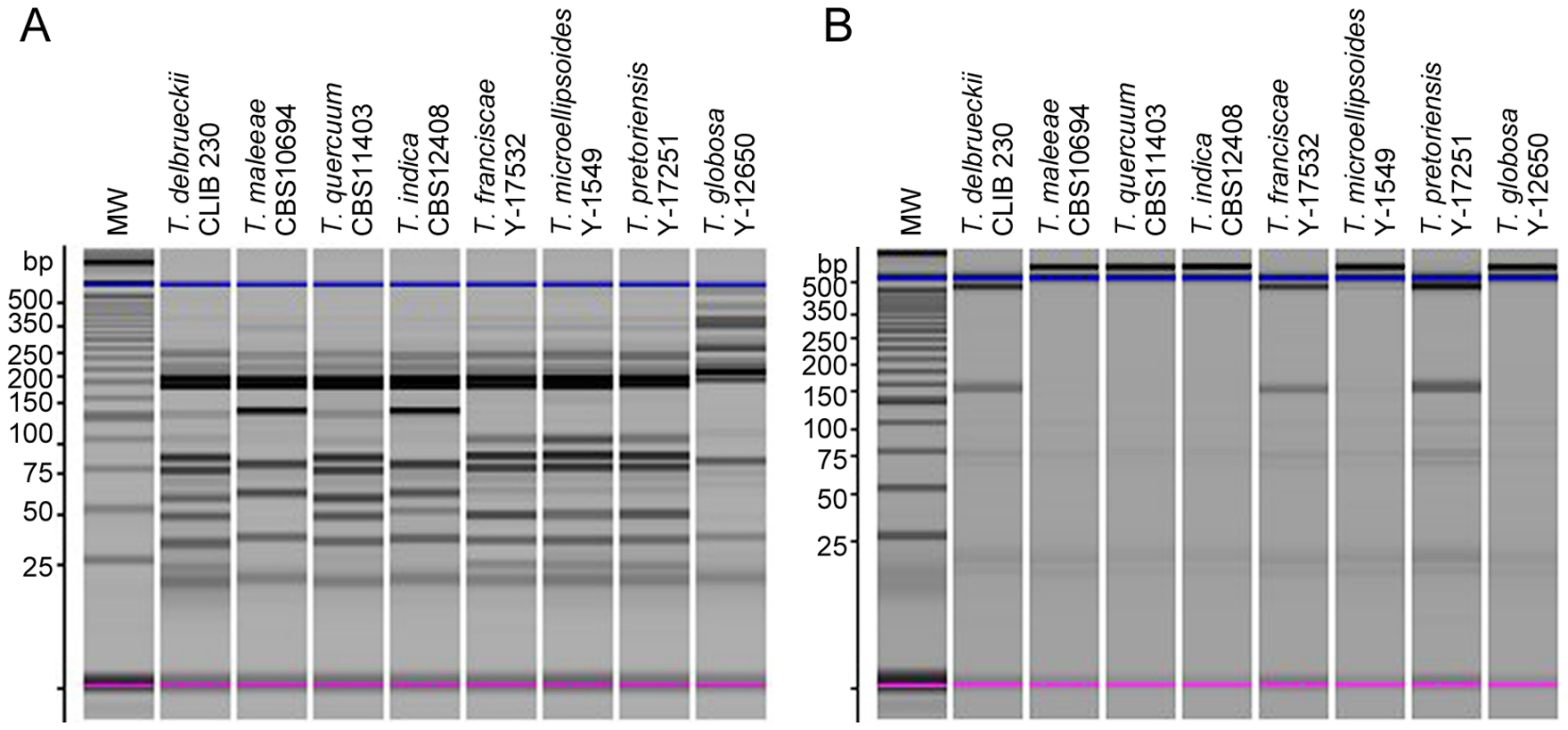
Restriction patterns of D1/D2 amplicon generated by *Alu*I (A) or *Pst*I (B) for *Torulaspora* species. A: For *Alu*I restriction, four patterns were produced: 170 pb+160 pb+80 pb+70 pb+55 pb+40 pb+30 pb for *T. delbrueckii* and *T. quercuum*; 170 pb+160 pb+120 pb+70 pb+55 pb+30 pb for *T. maleeae* and *T. indica*; 170 pb+160 pb+95 pb+80 pb+70 pb+30 pb for *T. franciscae*, *T. microellipsoides*, *T. pretoriensis*; and 330 pb+170 pb+75 pb+30 pb for *T. globosa*. B: For *Pst*I restriction, two patterns were produced: 600pb (no restriction) for *T. maleeae*, *T. quercuum*, *T. indica*, *T. microellipsoides* and *T. globosa*; or 480 pb+120 pb for *T. delbrueckii*, *T. franciscae* and *T. pretoriensis*. Blue and pink bands represent internal upper and lower markers respectively.

Enzymatic digestions of the 600 pb amplicon were carried out on 10 μl of amplified DNA in a final volume of 15 μl with either *Alu*I or *Pst*I (New England Biolabs, Ipswich, MA) for 16 h at 37°C. Restriction fragments were separated by a microchip electrophoresis system (MultiNA, Shimadzu). *Alu*I restriction allowed discriminating *T. delbrueckii* from all other species except *T. quercuum*, while *Pst*I digestion showed different restriction patterns for *T. delbrueckii* and *T. quercuum* (Figure 2).

### Microsatellite loci identification and primer design

Dinucleotide to tetranucleotide repeats were identified within the genome sequence of *T. delbrueckii* type strain CBS 1146^T^ (CLIB 230^T^) [59]. In order to exclude possible telomeric and subtelomeric repeats, we did not consider microsatellites located within 3Kb of the 5′-end or 3′-end of the contigs. Primers were designed using the ‘Design primers’ tool on the SGD website (http://www.yeastgenome.org/cgi-bin/web-primer). In order to reduce the cost associated with primer fluorescent labelling, the forward primers were tailed on the 5′-end with the M13 sequence (19 nt) as described by Schuelke, 2000 [60], allowing the use of M13 primers labelled with FAM or HEX for different PCR reactions. Amplified fragment sizes varied from ∼110 to ∼380 bp, allowing subsequent multiplexing of the amplicons (Table 1).

**Table 1:** Microsatellite loci for *Torulaspora delbrueckii* genotyping.

Microsatellite name	Motif	Primers	Fluorescent dye	Multiplex	Tm	Chromosome number and position (start:end)	Number of recorded alleles	Alleles size (repeats number) range	Allele size and repeats number for CLIB230^T^	Coding sequence
TD1A	CAA	F: AGATGCAACCACAATGGCAA; R: TGCGATTGAAACTGTTGATTG	HEX	M1	50	chromosome 1 (606452:606493)	25	161:269 (2:38)	187 (10)	hypothetical protein (TDEL0A03400)
TD1B	GT	F: TTCACAACTAGATGCCGATGT; R: TCCCGTCCTTCAAGTTAAACA	HEX	M2	51	chromosome 1 (511160:511201)	30	114:179 (2:35)	149 (20)	NA
TD1C	TTA	F: GTAAACATGTTTCGTAACGGG; R: CCTGGGATTCCATCCCAAT	HEX	M1	59	chromosome 1 (1056297:1056330)	23	327:379 (1:19)	357 (11)	NA
TD2A	GTT	F: GATGATGATGGTGATGCGAA; R: TCTTACAGAACTTTTCCCCGA	FAM	M1	51	chromosome 2 (1203158:1203120)	25	250:314 (4:25)	276 (13)	hypothetical protein (TDEL0B06790)
TD5A	GT	F: AGGGACCCCCACCTAAATTAA; R: CGAAAAAGTGAAACTACCTCGT	FAM	M1	51	chromosome 5 (450155:450190)	19	116:146 (5:20)	146 (20)	low-affinity hexose transporter (*LGT1*) gene
TD6A	CAA/CAG	F: AACAAGGGCTTATCATCCATT; R: ACCCCGCTTCTTTCTTCTTT	FAM	M2	55	chromosome 6 (751384:751430)	19	249:321 (0:24)	298 (16)	hypothetical protein (TDEL0F04060)
TD7A	TTAA	F: GAGGGAGTGGTACTATGGTGG; R: ACGCAGTGGTGTTCTTGAAT	HEX	M2	62	chromosome 7 (376649:376679)	6	231:263 (2:10)	252 (7)	NA
TD8A	TTG/CTG	F: AAATCAGTCGAGTAGGTTGCG; R: TCCACCGGGAATGTTCACT	FAM	M2	53	chromosome 8 (335387:335440)	40	133:245 (10:47)	156 (18)	hypothetical protein (TDEL0H01950)

Allele size in pb. Forward primers were tailed on 5′-end with M13 sequence (CACGACGTTGTAAAACGAC). Tm is the melting temperature used for microsatellite amplification (see Materials and Methods). CLIB230^T^ is synonymous of CBS 1146^T^.

### Microsatellite amplification

PCR reactions were performed in a final volume of 10 μl containing 50–100 ng of genomic DNA, 0.05 μM of forward primer, 0.5 μM of reverse primer and labelled primer, 1X Taq-&GO (MP Biomedicals, Illkirch, France). Universal primers M13 were labelled with either 6-carboxyfluorescein (FAM), or hexachlorofluorescein (HEX) (Eurofins MWG Operon, Les Ulis, France).

PCR was carried out using a thermal cycler (iCycler, Biorad, Hercules, CA, USA) as followed: Initial denaturation step (5 min at 95°C) followed by 35 cycles of 35 s at 95°C, 50 s at melting temperature (see Tm in Table 1) and 40 s at 72°C, and a final extension step of 7 min at 72°C.

Amplicons were initially analysed by a microchip electrophoresis system (MultiNA, Shimadzu) and the optimal conditions for PCR amplifications were assessed. Then, the sizes of the amplified fragments were measured on an ABI3730 DNA analyzer (Applied Biosystems). For that purpose, PCR amplicons were diluted (1800-fold for FAM and 600-fold for HEX-labelled amplicons respectively) and multiplexed (Table 1) in formamide. LIZ 600 molecular marker (ABI GeneScan 600 LIZ Size Standard, Applied Biosystem) was 100-fold diluted and added for each multiplex. Before loading, diluted amplicons were heated 4 min at 94°C. Allele size was recorded using GeneMarker Demo software V2.4.0 (SoftGenetics).

### Data analysis

Microsatellite analysis was used to investigate the genetic relationships between strains. A dendrogram was built using Bruvo's distance and Neighbor-Joining clustering, by means of the R program [61] and the following packages: polysat v1.3 [62], ape [63], adephylo [64], phyclust [65]. Bruvo's distance takes into account the peculiar mutational process of microsatellite loci, and is particularly well adapted for populations with mixed ploidy levels [66]. In order to assess the robustness of tree nodes, multiscale bootstrap resampling associated with an approximately unbiased test [67] was performed by means of R and the pvclust package v1.2-2 [61], [68].

In addition to dendrogram drawing, the software structure (v2.3.4) was used to delineate clusters of individuals on the basis of their microsatellite genotypes using a Bayesian approach [69]. Only strains with a maximum of two alleles per loci (*i.e.* considered as diploids) were conserved for structure analysis (104 strains upon 110). The parameters were as followed: 10000 Burn-in period, 1000 Repetitions. Models with number of populations (*K*) ranging from *K* = 3 to *K* = 20 were tested, and models with and without admixture gave similar results (the model with no admixture was thus conserved for the graphical representation of the population).

To test for population differentiation, analysis of molecular variance (AMOVA) was performed by means of the pegas package [70] with n = 1000 permutations. We tested whether the genetic distance was significantly explained by substrate origin and by geographical localisation (*i.e.* the continent of isolation was used as grouping factor). F-Statistics (*F_ST_, F_IT_, F_IS_*) were computed for each locus using Weir and Cockerham formula [71], and we tested whether the genotype frequencies of each locus followed the Hardy–Weinberg equilibrium by means of the pegas package [70].

In order to obtain an estimate of the divergence time between different *T. delbrueckii* genetic clusters from the microsatellite data, we used the method described by Goldstein et al. [72]. In yeasts, the mutation rate for microsatellites generally falls between 1.10^−4^ and 1.10^−6^ per cell division [73], [74], so we used an average mutation rate of 1.10^−5^ per cell division. The number of generations per year in yeast populations is difficult to estimate correctly: Fay and Benavides considered a maximum of 2920 generations per year for domesticated *S. cerevisiae* [75], but this may be considerably lower for wild yeast populations [76] for which we considered a 100 generations per year as a maximum.

## Results

### Development of polymorphic microsatellite markers for *Torulaspora delbrueckii*

We took advantage of the recent *de novo* assembly of the genome sequence of CBS 1146^T^ (synonymous to CLIB 230^T^) [77], the type strain of *Torulaspora delbrueckii*, to search for microsatellite loci. We only considered dinucleotide to tetranucleotide repeats that were not located within the 5′-end and 3′-end of the chromosomes, in order to exclude possible telomeric or subtelomeric positions. We retained eight microsatellite loci that were located on six of the eight chromosomes of CBS 1146^T^ (Table 1). Some loci were located in coding regions, like TD5A (low-affinity hexose transporter *LGT1* gene) or TD1A, TD2A, TD6A and TD8A, located in hypothetical protein coding sequences. The three remaining loci, TD1B, TD1C and TD7A were in non-coding regions.

The amplicons were separated using a microchip electrophoresis system (MultiNA), and the optimal conditions for microsatellites amplifications were assessed on a panel of twenty strains of *T. delbrueckii* (data not shown). After optimisation on *T. delbrueckii* strains, the microsatellites markers were tested on seven additional species of the *Torulaspora* genus: *T. franciscae, T. pretoriensis, T. microellipsoides, T. globosa, T. indica* [54], *T. maleeae* [55], and *T. quercuum* [56]. All these species appear to produce good amplification of several markers, suggesting that some of the microsatellites developed for *T. delbrueckii* could be useful for the study of population genetics of other *Torulaspora* species. In particular, the type strain of *T. pretoriensis* Y-17251^T^ showed a complex genotype, with three alleles for three loci (TD1B, TD2A, TD6A), two alleles for three loci (TD1A, TD5A, TD8A) and one allele for the remaining two loci (TD1C, TD7A). This suggested *T. pretoriensis* Y-17251^T^ could be aneuploid or polyploid. Further characterization of additional *T. pretoriensis* strains will help determine whether all strains share complex genome or whether complex genotype is strain-specific.

The eight microsatellites markers were then used to genotype 110 *T. delbrueckii* strains isolated from worldwide regions (Figure 1, Figure S1) and from various substrates (Table S1): 34 strains were natural isolates isolated from plants, insects, soil, etc., 36 strains were associated with several bioprocesses including bakery, cider brewery, dairy processes, other fermented beverage and food industries, excluding winemaking. While winemaking is a bioprocess, a particular focus was placed on strains from grape/wine habitats due to long historical association between winemaking and *T. delbrueckii*, in comparison to other bioprocesses. Thus, 35 strains related to winemaking and isolated from grapes, must, wine or oenological material, were included. Finally, 3 clinical strains were also included in the collection, as well as 2 strains of unknown origin (one of which was the type strain CBS 1146^T^  =  CLIB 230^T^). All strains were able to grow on minimal medium (SD) and YPG medium containing glycerol as the sole source of carbon, indicating that all 110 strains were prototrophs and able to respire. In addition, all 110 strains studied here were able to sporulate and usually form ascii with one to four ascospores, ascii with a single ascospore were far more frequent than those with 2 to 4 ascospores.

Genotyping the 110 strains of our collection revealed that all microsatellites were polymorphic, with 6 different alleles for TD7A and up to 40 alleles for TD8A (Table 1, Table S2). For some loci, some strains showed allele size incompatible with the stepwise mutation model predicting the increase or decrease in repetition number and thus strict motif multiplication [78]. This indicated that some punctual insertion/deletion may arise either within the microsatellite locus itself, thus increasing motif complexity as previously shown [79], [80], or within adjacent amplified sequence. Finally, all 110 strains tested showed unique genotype, confirming the discriminant power of microsatellite analysis.

### Human activities shaped the genetic variability of *Torulaspora delbrueckii* species

**Figure 3:**
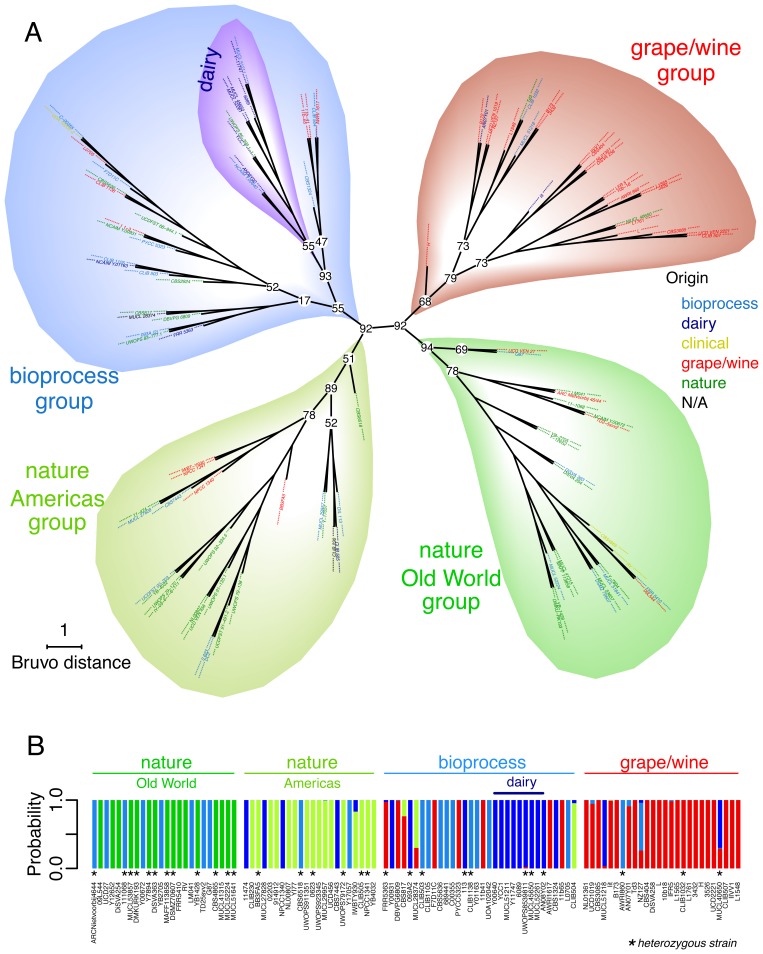
Genetic relationships between 110 *T. delbrueckii* strains using eight microsatellite markers. A: Dendrogram tree built using Bruvo's distance and Neighbor-Joining's clustering. The robustness of the node was assessed using multiscale bootstrap resampling and approximated unbiased test (n = 1000 boots). Bootstrap results are shown only for the main nodes. B: Barplot representing structure results (K = 5). The posterior probability (y-axis) of assignment of each strain (vertical bar) to ancestral groups is shown by colors (dark green, green, blue, red and darkblue colors represent each 5 ancestral populations). Heterozygous strains, meaning strains with at least one heterozygote locus, are indicated by black stars.

The genetic relationships between the 110 strains of *T. delbrueckii* were further examined using Bruvo's distance (which is particularly well-adapted for microsatellite data and populations with unknown/variable ploidy levels) and Neighbor-Joining clustering. The resulting dendrogram tree showed four main clusters that were strongly related to substrate origin (Figure 3A). Two groups contained most strains isolated from nature, with a clear dichotomy depending on their geographical origin. Indeed, “nature/Americas” group comprised 25 strains of which 12 were isolated from plants, insects or soils, and 16 isolated on the American continent (with representatives from North, Central and South America). The nature/Americas group was moderately supported with bootstrap value of 51, due to the uncertain position of CBS6518 strain. The descending node (excluding CBS6518) was much more robust (bootstrap value of 89). The “nature/Old World” group comprised 24 strains, 12 out of 24 were indeed isolated from nature (plants and soils), and 18 out of 24 were isolated from the Old World (Europe, Asia and Africa). Nature/Old World group was very robust (bootstrap values of 94).

The third cluster, designed as “grape/wine” group, was composed of 27 strains, most of them (21/27) being isolated from grapes or wines. The grape/wine group was moderately supported (bootstrap value of 58), due to uncertain position of H strain. However, inferior node (excluding H strain) was much more robust (79). Interestingly, this group was not structured according to the geographical origin, and the main wine regions of the world were all represented (several European regions, California and South America, Australia and New Zealand). The last group was moderately supported (bootstrap value of 55) and contained 34 strains, of which 18 were associated with various bioprocesses and human activities (excluding winemaking) from the five continents. Noticeably, within this so-called “bioprocess” group, a sub-cluster containing mostly dairy strains was observed (6 of 11 strains), with a moderately supported bootstrap value (55). Analysis with the program structure was congruent with the dendrogram tree: structure found an optimum of *K* = 5 populations that captured the major genetic structure of *T. delbrueckii* species (Figure 3B). These populations were consistent with the four genetic clusters previously defined from the dendrogram tree (nature/Americas, nature/Old World, grape/wine and other bioprocesses), and also supported the dairy group (Figure 3B).

In order to definitively determine whether, and to what extent, the genetic variation of *T. delbrueckii* was related to substrate and/or geographical origin, an analysis of molecular variance (AMOVA) was performed. We used either the substrate origin (nature, bioprocess, clinical, grape/wine), or the geographical localisation (using continent of isolation) as grouping factors. The substrate origin explained 12.29% of the total variation of microsatellite dataset, and was strongly significant (p-value<0.00001), indicating that substrate origin shaped significantly, yet not completely, the *T. delbrueckii* population structure. By contrast, the geographical localisation, although significantly related to molecular variation (p-value<0.001), explained less variation (7.61%). It should be noted that the geographical localisation, when considering only strains from nature, explained 17.19% of the genetic variation of nature isolates (and was significant with p-value<0.001), while it was no longer significant when considering strains from bioprocess and grape/wine origins. This confirmed that human activities, namely winemaking or other bioprocesses, significantly shaped the genetic variability of the corresponding *T. delbrueckii* strains, while nature isolates are differentiated on the basis of geographical localisation, as expected for a wild population.

### *Torulaspora delbrueckii* is a highly inbred species

Individually, the 110 *T. delbrueckii* strains included in the analysis had one to 4 alleles per locus. Most strains (71 out of 110) were homozygous for all eight loci, while 33 out of 110 presented a maximum of two alleles per locus. Five strains (B172, CBS2924, DC2, DIL 113 and UWOPS 79-138) had a maximum of 3 alleles, but for one locus only, the hallmark of punctual genetic duplication or aneuploidy rather than whole genome duplication (polyploidy). Finally, UWOPS 83-777.1 had 3 and 4 alleles for TD1A and TD5A respectively, suggesting a more complex genome structure.

Considering *T. delbrueckii* to be a diploid species, we calculated different population parameters (Table 2). Observed heterozygosity (Ho) was low, from 0.009 for TD7A (the less polymorphic microsatellite locus) to 0.128 for TD1C and TD5A (mean Ho = 0.087). Indeed, the population data strongly deviated from Hardy-Weinberg expectations for all 8 microsatellite loci, with excess in homozygous strains (Table 2). This was particularly evident for the “grape/wine” group, with only three out of 27 heterozygous strains (Figure 3). To determine whether such high level of homozygosity was due to inbreeding and/or to subpopulation differentiation, we calculated F-statistics (*F_ST_*, *F_IS_* and *F_IT_*, Table 2). All 8 loci gave similar results: a high total deficit of heterozygotes (*F_IT_*), *i.e.* close to 1, associated with high deficit of heterozygotes within the population (*F_IS_*), indicating that the excess of homozygote individuals was mainly due to high inbreeding within each subpopulation rather than to subpopulation differentiation.

**Table 2:** F-statistics and observed heterozygosity in *Torulaspora delbrueckii* population.

Microsatellite	*F_IT_*	*F_ST_*	*F_IS_*	Ho
TD1A	0.914	0.034	0.911	0.103 ***
TD1B	0.906	0.104	0.896	0.120 ***
TD1C	0.877	0.020	0.874	0.128 ***
TD2A	0.945	0.118	0.938	0.077 ***
TD5A	0.861	0.162	0.834	0.128 ***
TD6A	0.972	0.140	0.968	0.034 ***
TD7A	1.00	0.065	1.00	0.009 ***
TD8A	0.921	0.050	0.917	0.094 ***

*F_IT_* represents the total deficit of heterozygotes, *F_IS_* the deficit of heterozygotes within the population, *F_ST_* the fixation index. *** indicates a significant effect at 0.1%. Ho stands for observed heterozygosity, and did not fit the Hardy-Weinberg hypothesis (pval<<0.001) for all eight loci.

### Estimating the divergence time of *Torulaspora delbrueckii* genetic groups

We estimated the divergence time between the different *T. delbrueckii* genetic clusters from microsatellite data using the method described by Goldstein et al. [72]. We used an average mutation rate of 1.10^−5^ per cell division, as the mutation rate for microsatellite in yeast generally falls between 1.10^−4^ and 1.10^−6^ per cell division [73], [74]. The number of generations *per* year in wild yeast populations is difficult to estimate correctly [76], but we considered a 100 generations per year as a maximum. Indeed, in winemaking and most bioprocesses, the population size of indigenous *T. delbrueckii* completed 7 generations *per* process [81], which can be repeated a few times *per* year, so that 100 generations *per* year seems to be a maximum. For wild strains, knowing that yeast growth requires a combination of favourable physico-chemical conditions as well as nutrient availability (sugar, nitrogen), we assumed wild strains would only grow during spring/summer which may limit their growth to less than one hundred generations a year. Using these parameters, the grape/wine cluster was estimated to diverge from the nature/Old World group 1908 years ago [95% interval confidence: 1233–2125 years ago]. By contrast, the bioprocess group was older and diverged from the nature/Americas cluster 3882 years ago [95% interval confidence: 2961–5671 years ago].

## Discussion

### *Torulaspora delbrueckii* is a domesticated species for winemaking and bioprocesses

Microsatellite genotyping is widespread for population, ecological and evolutionary studies of eukaryote species [82], [83], and provided new insights into the population structure of *S. cerevisiae* yeast [5], [84]–[86]. In particular, the strong relationship between genetic clustering and biotechnological applications [5] indicated that *S. cerevisiae* was a domesticated species. The present study shows that *T. delbrueckii* strains also cluster depending on their human use, with the existence of genetic groups connected to winemaking and bioprocesses. Many groups had high bootstrap values, the likeliness of the four groups was further confirmed by structure analysis, and the relationship between genetic variation and substrate origin was statistically validated by AMOVA.

The “grape/wine” group is particularly interesting, with strains from various continents demonstrating more genetic proximity than strains from the same continent but from different substrates (bioprocess, nature). This suggests that a group of closely related individuals gave rise to the “grape/wine” population, and were selected, consciously or unconsciously, for wine production. For *S. cerevisiae*, the wine domestication event occurred 10 000–12 000 years ago (coinciding with the first archaeological records of winemaking), indicating that *S. cerevisiae* was selected at the very beginning of wine production [5]. To determine if the wine domestication of *T. delbrueckii* preceded that of *S. cerevisiae*, the divergence time between the different *T. delbrueckii* genetic clusters was estimated from microsatellite data. The grape/wine cluster diverged from nature/Old World group around 1900 years ago, suggesting that *T. delbrueckii* domestication for winemaking is much more recent than *S. cerevisiae* and is related to the modern history of oenology. More precisely, *T. delbrueckii* domestication is contemporary with the Roman Empire where *Vitis vinifera* expanded throughout Europe [87]. In the Middle Ages, *V. vinifera* further expanded throughout the Old World with religions extension, *i.e.* Christian crusades in Northern Europe, Islam spread to North Africa and Middle East [87]. As *T. delbrueckii* is frequently isolated on grapes and other plants, we hypothesized that an ancestral “grape/wine” population of *T. delbrueckii* spread all around the world with grapevines varieties during the Roman Empire and the Middle Ages, and that their progeny was thus associated with vinification practices in the different wine regions.

Remarkably, besides its use for winemaking, *T. delbrueckii* strains are associated with several other human activities (dairy products, bakery, distillery, other food and beverage fermentation) that clustered together. The bioprocess group was estimated to be older than *T.delbrueckii* domestication for wine and to date back around four millennia ago, suggesting simultaneous anthropization of *T. delbrueckii* and *S. cerevisiae* for several food and beverage processes during the Neolithic era [88]. Within the bioprocess group, a sub-specialization was observable for dairy products, like *S. cerevisiae* for which specialization for beer, bread or sake was reported [75], [88]. In addition to the grape/wine and bioprocess clusters, we identified two groups mostly containing strains from nature, indicating that *T. delbrueckii* consists of both wild and domesticated populations, as shown for *S. cerevisiae* [75].

Although microsatellite analysis showed strong evidence for wine and bioprocess domestication, the genetic clustering was not perfect, suggesting frequent exchanges between subpopulations that may be mediated by insects or human activities as suggested for *S. cerevisiae* [86], [89]. To date, *T. delbrueckii* is the only non-Saccharomyces yeast proved to be domesticated and thus constitutes a complementary model system to the *Saccharomyces* genus. Population genetics of other yeasts of biotechnological interest will help determine whether anthropization shaped significantly the genetic variability of various yeasts besides *S. cerevisiae* and *T. delbrueckii*.

### Understanding the life-cycle of *Torulaspora delbrueckii*

In addition to population structure, microsatellite analysis may be useful for understanding the life-cycle [90]. Different life cycles could be congruent with the results presented here, the main ones being either a mostly diploid life-cycle, with both homozygous and heterozygous homothallic diploid representatives, and a few aneuploid/polyploid individuals; or a mostly haploid life-cycle, with both haploid (homozygous) and diploid (heterozygous) heterothallic individuals, as well as a few aneuploid/polyploid individuals.

*T.delbrueckii* was formerly described as a haploid species [91]–[93], because of its small cell size and also because tetrads are rarely observed following sporulation. However, several lines of evidence suggest that *T. delbrueckii* may not be haploid: first, the recent genome sequencing of type strain CBS 1146 reveals that, at the genetic level, *T. delbrueckii* possesses apparently functional mating-type (*MAT*) locus and silent *HMR* and *HML* loci, suggesting this species could be homothallic [77]. Secondly, all 110 strains we studied here (homozygous and heterozygous) were able to sporulate and mostly formed ascii with a single ascospore. Strains isolated under the name of *Candida colliculosa*, that should theoretically represent the anamorphic forms, displayed similar sporulation abilities to their so-called teleomorph counterparts. In particular, the clinical isolates showed the same sporulation characteristics as their non-clinical counterparts and were distributed on the dendrogram tree, indicating that *T. delbrueckii* is an opportunistic pathogen rather than an actual human pathogen. It has to be noted that all *T. delbrueckii* strains sporulated on traditional sporulation (acetate) medium after 3 days, but also after 7–20 days on YPD-agar plates, indicating that, unlike *S. cerevisiae*, starvation is not necessary for sporulation [94]. Thirdly, our genotyping data are in accordance with the hypothesis that it is a diploid species (with the identification of 30% of heterozygous individuals), associated with frequent inbreeding and thus frequent diploid homozygous individuals (65%). Such high inbreeding could be explained by the effect of homothallism on population genetic structure, furthermore enhanced by the aptitude of *T. delbrueckii* to sporulate without starvation. The occasional identification of strains with three or four alleles for a few loci suggests either a gene duplication ability, aneuploidy or even polyploidy, as for *S. cerevisiae* [5], [95]. All these results suggest a life cycle identical to the homothallic diploid *S. cerevisiae* yeast. Further experiments, like micro-dissection and genetic analysis of *T. delbrueckii* monosporic clones, construction of haploid heterothallic strains, etc., will elucidate definitively the life-cycle of *T. delbrueckii* species.

### Toward genetic improvement of *T. delbrueckii* species for industrial purpose

Species with biotechnological interest are usually improved for industrial purpose, through selection experiments, breeding programs, QTL detection, etc. This is the case of the yeast *Saccharomyces cerevisiae* for which several improvement programs are running for different technological applications (winemaking, bakery, brewery, distillery, etc.) [96], [97].

Among the non-conventional yeasts naturally associated with food processes, *T. delbrueckii* is particularly interesting: in winemaking, *T. delbrueckii* allows reducing organoleptic defects (hydrogen sulphide, volatile phenols, volatile acidity) [16], [17] and increases sensorial complexity [18]–[20]. Thus, recently, several strains of *T. delbrueckii* were commercialized for winemaking purpose with success, to be used in association with *S. cerevisiae*. Besides winemaking, microsatellite analysis provides evidence of anthropic selection for other bioprocesses, and a possible specialization for dairy process.

Here, microsatellites markers were developed and gave valuable data regarding the life-cycle of the species, suggesting a mostly diploid homothallic life. A better understanding of life-cycle and the availability of highly discriminant markers paves the way toward further biotechnological improvement of *T. delbrueckii* strains for winemaking and other bioprocesses purposes.

## Supporting Information

Figure S1 **European localisation of the** ***T. delbrueckii*** **strains used in this study.**
(PDF)

Table S1 **Origin of** ***Torulaspora spp.*** **strains used in this study.** ^a^ ARC-INFRUITEC: Agricultural Research Council-Institute for Deciduous Fruit, Vines and Wine; ARS Culture Collection: Agricultural Research Service Culture Collection, formerly NRRL (Northern Regional Research Laboratory); AWMCC: AWRI Wine Microorganism Culture Collection; BCCM/MUCL: Agroindustrial fungi & yeasts collection, Mycotheque de l'Universite catholique de Louvain; CBS-KNAW: Centraalbureau voor Schimmelcultures (CBS) Fungal Biodiversity Centre, institute of the Royal Netherlands Academy of Arts and Sciences (Koninklijke Nederlandse Akademie van Wetenschappen); CIATEJ: Centro de Investigación y Asistencia en Tecnología y Diseño del Estado de Jalisco; CIRM-Levures: Centre International de Ressources Microbiennes –Levures, formerly CLIB: Collection de Levures d'Intérêt Biotechnologique; CRB Oeno: Centre de Ressources Biologiques Œnologie; CRPR: Centre de Recherche Pernod-Ricard; DIL: Deutsches Institut fur Lebensmitteltechnik e.V.; D.i.S.V.A.: Dipartimento di Scienze della Vita e dell'Ambiente; DSMZ: Deutsche Sammlung von Mikroorganismen und Zellkulturen GmbH; IDEPA: Instituto Multidisciplinario de Investigación y Desarrollo de la Patagonia Norte; IFI: Instituto de Fermentaciones Industriales; IFV: Institut Français de la Vigne et du Vin; ISVV: Institut des Sciences de la Vigne et du Vin; IWBT: Institute for Wine Biotechnology; LYCC: Lallemand Yeast Culture Collection; MRI: Max Rubner-Institut; NCAIM: National Collection of Agricultural and Industrial Microorganisms; NIAS: National Institute of Agrobiological Sciences; NPCC: North Patagonian Culture Collection; PYCC: Portuguese yeast Culture Collection; UOA/HCPF: Hellenic Collection of Pathogenic Fungi, University of Athens; UWOPS: Culture collection of the University of Western Ontario, Department of Biology (formerly Plant Sciences). ^b^ Grant FONDEF D98I1037- Chile. Collection and characterization of native yeast strains for differentiation and identity of Chilean wine (1999-2003).
(XLSX)

Table S2 **Genotype of 110 strains of** ***Torulaspora delbrueckii*** **using eight microsatellite markers.** For each strain and each marker, the size of the amplicons is indicated. For heterozygous loci, the different alleles are separated by a slash. NA stands for “Not Available” (missing) data.
(XLSX)
