# Supplementary figures and images for: Winemaking and Bioprocesses Strongly Shaped the Genetic Diversity of the Ubiquitous Yeast Torulaspora delbrueckii
Source: PLoS One. 2014 Apr 9;9(4):e94246. doi: 10.1371/journal.pone.0094246 (PMC3981792; doi:10.1371/journal.pone.0094246)

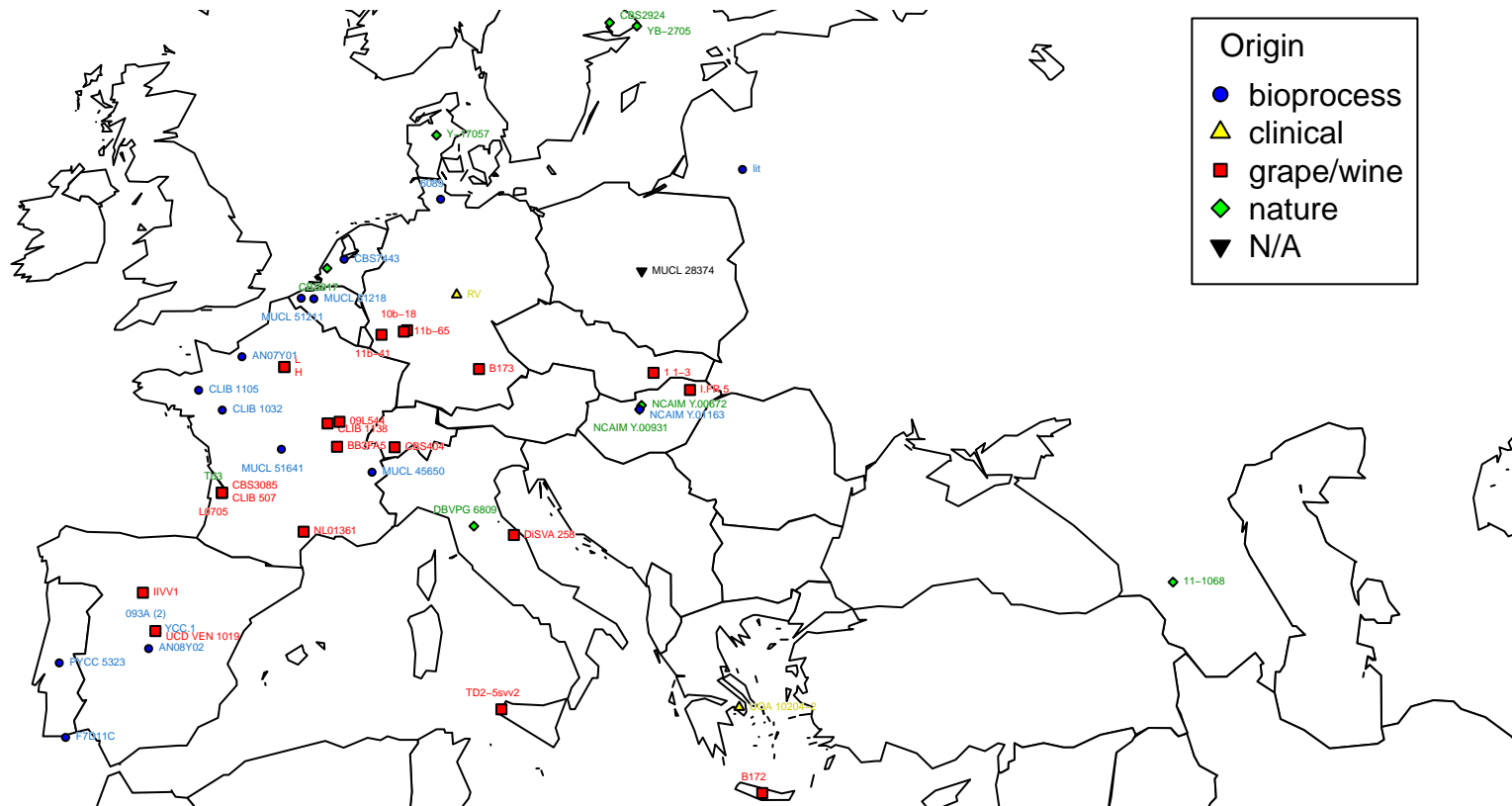

Supplement: Figure S1 — European localisation of the T. delbrueckii strains used in this study. (PDF) [file pone.0094246.s001.pdf]
